# Golden Gate assembly with a bi-directional promoter (GBid): A simple, scalable method for phage display Fab library creation

**DOI:** 10.1038/s41598-020-59745-2

**Published:** 2020-02-19

**Authors:** Karuppiah Chockalingam, Zeyu Peng, Christine N. Vuong, Luc R. Berghman, Zhilei Chen

**Affiliations:** 1 Department of Microbial Pathogenesis and Immunology, Texas A&M University Health Science Center, College Station, Texas 77843 USA; 2 Department of Poultry Science, Texas A&M University, College Station, Texas 77843 USA; 3 Present Address: Biosion, Inc., Nanjing, 210061 China; 4 Present Address: Department of Poultry Science, University of Arkansas, Fayetteville, Arkansas 72703 USA

**Keywords:** High-throughput screening, Antibody therapy

## Abstract

Fabs offer an attractive platform for monoclonal antibody discovery/engineering, but library construction can be cumbersome. We report a simple method – Golden Gate assembly with a bi-directional promoter (GBid) – for constructing phage display Fab libraries. In GBid, the constant domains of the Fabs are located in the backbone of the phagemid vector and the library insert comprises only the variable regions of the antibodies and a central bi-directional promoter. This vector design reduces the process of Fab library construction to “scFv-like” simplicity and the double promoter ensures robust expression of both constituent chains. To maximize the library size, the 3 fragments comprising the insert – two variable chains and one bi-directional promoter – are assembled via a 3-fragment overlap extension PCR and the insert is incorporated into the vector via a high-efficiency one-fragment, one-pot Golden Gate assembly. The reaction setup requires minimal preparatory work and enzyme quantities, making GBid highly scalable. Using GBid, we constructed a chimeric chicken-human Fab phage display library comprising 10^10^ variants targeting the multi-transmembrane protein human CD20 (hCD20). Selection/counter-selection on transfected whole cells yielded hCD20-specific antibodies in four rounds of panning. The simplicity and scalability of GBid makes it a powerful tool for the discovery/engineering of Fabs and IgGs.

## Introduction

The discovery and engineering of monoclonal antibodies (mAbs) using phage display usually utilizes one of two simplified display formats. Single-chain variable fragments (scFvs) represent the simplest format for antibody phage display in which only the domains directly involved in interaction with the antigen – the variable heavy and variable light chains – are represented. Antibody-binding fragments (Fabs) more closely resemble the antigen-binding “business end” of IgG molecules and incorporate both the variable regions and their immediately neighboring constant subdomain – typically CH1 for the heavy chain and CLκ or CLλ for the light chain. Under oxidizing conditions, the constant domains in Fabs are covalently linked via a disulfide bond.

The attractiveness of the scFv as a medium for IgG discovery lies in its overall simplicity. The presence of only a single chain generally gives rise to higher expression, and cloning of scFvs into phagemid vectors is relatively straightforward, typically requiring only a single overlap extension PCR reaction that links the two variable domains via a short flexible linker^1–3^. Unfortunately, the binding affinity of scFvs can change significantly upon reformatting to Fabs or IgGs, creating a need to verify the binding/activity of the molecules in the more complex formats in applications where Fabs or IgGs are the desired final product^4–8^.

Fabs more closely resemble full-length IgGs and are generally more stable than scFvs^6,9^, making these molecules a more reliable indicator of full-length IgG behavior. Unfortunately, the construction of phage display Fab libraries is less straightforward than scFv libraries, requiring independent expression of two fusion constructs – a heavy chain and light chain, each comprising a variable and constant domain. Expression of the two chains is conventionally achieved via a single transcript containing separate ribosome-binding sites for each chain^10–13^. Interestingly, although the single-transcript configuration has been widely used, the approach of using a single-promoter bicistronic expression cassette tends to yield much lower expression of the gene positioned furthest from the promoter relative to a two-promoter system^14^. Construction of the Fab expression cassette typically involves sequential overlap extension PCR reactions that tend to yield significant non-specific products manifesting as smears in agarose gel electrophoretic analysis^13,15^. Moreover, the need to PCR-amplify the constant domains creates the risk of introducing unwanted point mutations into these regions.

**Figure 1:**
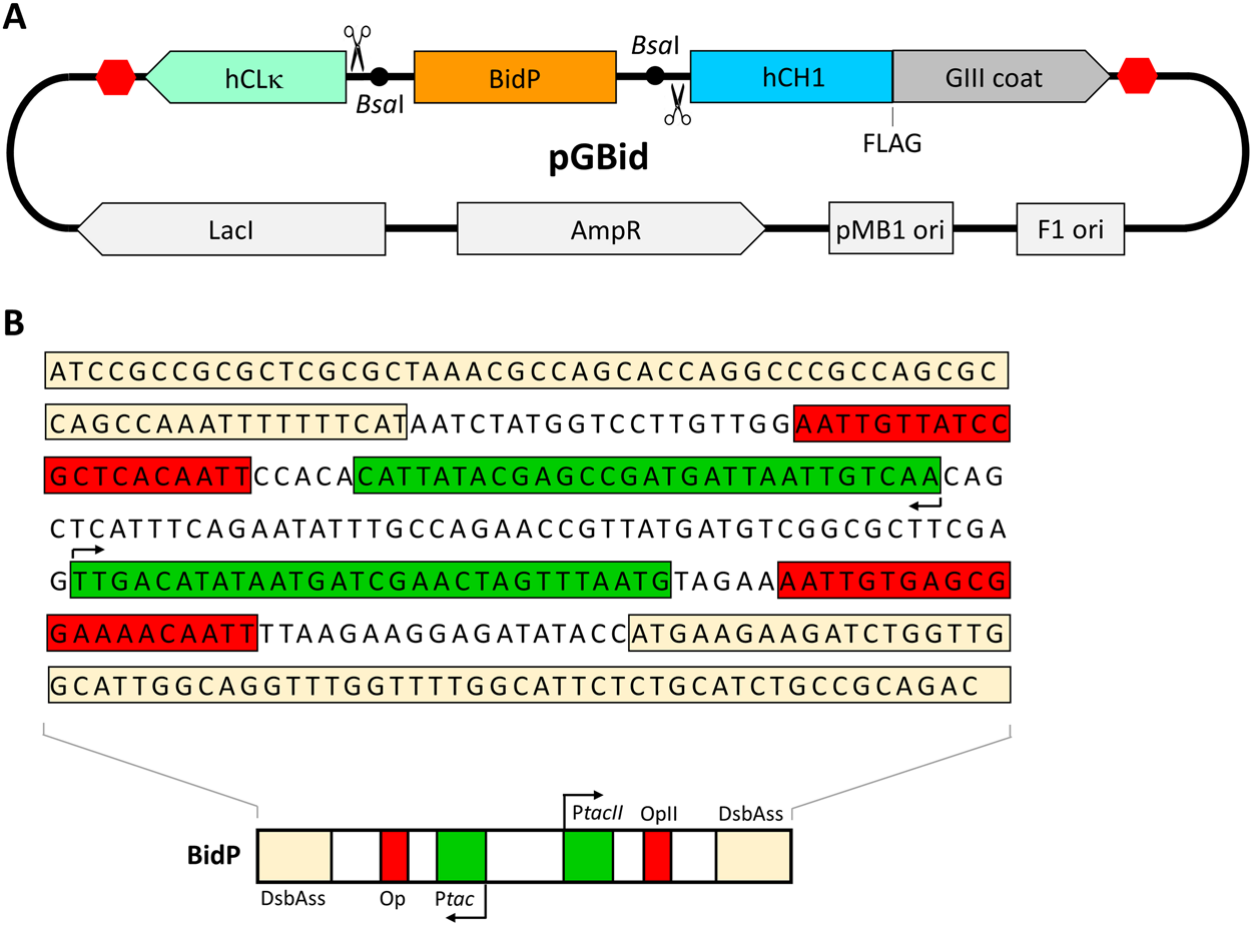
GBid-mediated Fab display on phage. (**A**) Schematic of phagemid pGBid. The red hexagons represent terminator sequences. (**B**) Design of the bi-directional promoter cassette, BidP. P*tac* controls the expression of the light chain, and P*tac*II the heavy chain. Op, lac operator; OpII, modified lac operator with weakened affinity for the lac repressor; DsbAss, DsbA signal sequence.

We sought to simplify the process of phage display Fab library creation by making three key design modifications to a “standard” M13 P3 coat protein display phagemid (Fig. 1A). First, we incorporated the constant domains of the Fabs, specifically the CLκ and CH1 domains, into the backbone of the phagemid, obviating the need to amplify these domains during cloning. Second, we introduced recognition sites for the Type IIS restriction enzyme *Bsa*I immediately upstream of the N terminus of both constant domains to facilitate seamless linkage of the variable fragment via “one-pot” Golden Gate assembly^16^. Third, we designed a bi-directional promoter based on the *tac* series of promoters^17^ to drive the robust expression of both the light and heavy chains. Immediately flanking the bi-directional promoter (BidP) and included as part of the BidP cassette are sequences encoding a DsbA signal peptide directing protein expression to the cotranslational SRP pathway^18^.

Our new phagemid vector, pGBid, enables creation of Fab libraries via two simple steps – (*i*) a 3-fragment overlap extension PCR reaction to combine the central BidP with the two flanking variable domains, and (*ii*) a one-pot, one-fragment high-efficiency Golden Gate assembly reaction requiring only minimal amounts of enzyme to seamlessly incorporate the library insert into pGBid, without the need for separate restriction digestion or ligation reactions or agarose gel extraction of the input DNA. This simple and scalable approach to the creation of Fab-displaying phage, referred to henceforth as Golden Gate with a bi-directional promoter (GBid), was validated by the incorporation of a functional HER2-binding Fab on the surface of M13 bacteriophage. More importantly, we demonstrate the use of GBid to create and select a large Fab phage display library with variable domains sourced from chickens immunized with cells displaying the complex 4-transmembrane domain protein human CD20 (hCD20). The isolation of hCD20-specific mAbs from this library following four rounds of selection/counter-selection on transfected whole mammalian cells underscores the value of GBid for Fab/mAb discovery, even for difficult targets.

## Results

### Phage display of Fabs via GBid

The phagemid pGBid was constructed as depicted in Fig. 1. The CH1 domain of human IgG1, hCH1, was placed upstream of the C-terminal domain of the M13 bacteriophage GIII minor coat protein in a phagemid^19^. The human CL-kappa domain (hCLκ) followed by a synthetic terminator sequence was positioned adjacent to the hCH1 domain in the opposite direction. An isopropyl β-D-1-thiogalactopyranoside (IPTG)-inducible bi-directional promoter cassette named BidP, composed of two opposite-facing *tac* promoters flanked by a pair of DsbAss cotranslational translocation signal peptides^18^, was inserted between the hCH1 and hCLκ domains. Recognition sites for the Type IIS restriction enzyme *Bsa*I were included immediately upstream of the constant domains to enable seamless Golden Gate-mediated incorporation of a VL-BidP-VH cassette during Fab library construction. We note that the bi-directional promoter cassette BidP is included in this vector only as a template for PCR amplification, as the BidP cassette contained in the vector is removed during Golden Gate incorporation of the VL-BidP-VH insert.

The sequences of the constituent promoters in BidP – P*tac* and P*tac*II – are derived from ref. ^17^. The use of different constituent promoters minimizes the likelihood of DNA instability caused by the presence of repetitive sequences. Since P*tac*II is reportedly a weaker promoter than P*tac*, a compensatory T→A base substitution was made within the lac operator (OpII, Fig. 1B) downstream of P*tac*II to weaken its interaction with the lac repressor^20^. A 51-base spacer was inserted between P*tac* and P*tac*II to allow adequate room for RNA polymerase-mediated transcription initiation in both directions. In the current configuration of GBid, P*tac* controls the expression of the VL-CLκ chain and P*tac*II controls VH-CH1 chain expression, although we anticipate that the BidP cassette should also work if it is flipped and the constituent *tac* promoters reassigned to different chains.

**Figure 2:**
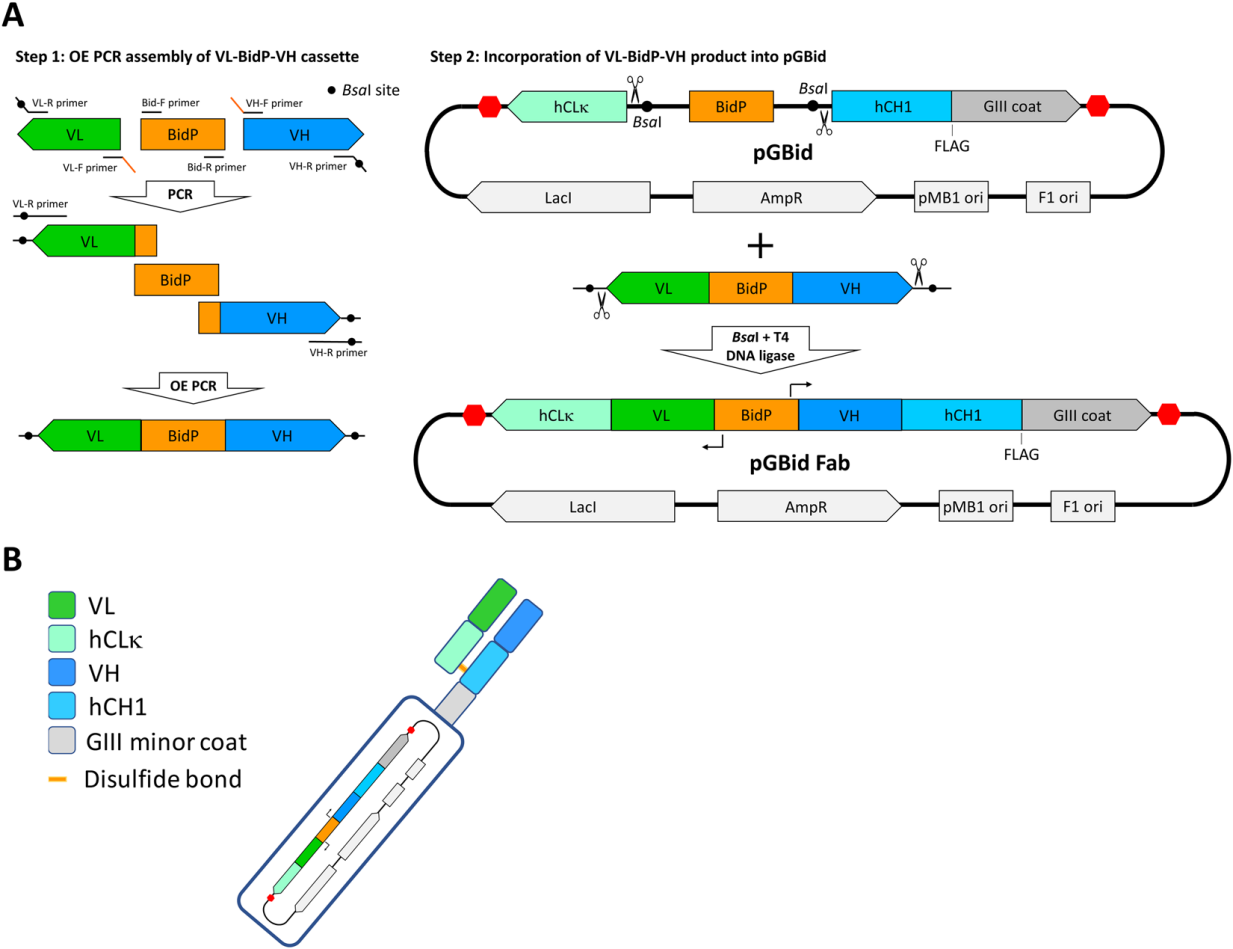
Two-step GBid cloning of Fabs into pGBid. (**A**) Step 1: construction of the VL-BidP-VH cassette via a 3-fragment overlap extension PCR. Sequences of primers are presented in Table 1. Step 2: incorporation of the VL-BidP-VH product into pGBid via Golden Gate assembly. (**B**) Schematic of Fab displayed on the surface of M13 bacteriophage.

The display of Fabs on the M13 phage P3 coat protein is achieved via a simple two-step process (Fig. 2). First, the VL, VH, and BidP fragments are amplified from the relevant templates via high-fidelity PCR and assembled via a three-fragment overlap extension PCR. The primer design for the amplification of the VL and VH fragments is dependent on the source/nature of the variable fragments. Examples of primers used for VL and VH amplification in this study are presented in Table 1. BidP is amplified from pGBid using primers Bid-F and Bid-R (Table 1). In the second step of GBid, the assembled VL-BidP-VH fragment is cloned into pGBid via a high-efficiency one-fragment, one-pot Golden Gate assembly reaction.

**Table 1:** Primers used for GBid-mediated cloning in this study. *Bsa*I recognition sites are underlined.

Primer ID	Sequence (5′ → 3′)
chVL-F	CGAGCGCGGCGGATGCGCTGACTCAGCCGTCC
chVL-R	TCTGCAGGTCTCATACGTAGGACGGTCAGGGTTGTCCC
chVH-F	CATTCTCTGCATCTGCCGCAGACGCGGTGACGTTGGACGAG
chVH-R	AGGTCTGGTCTCTAAGCGGAGGAGACGATGACTTCGGTC
Trast VL-F	AGCGCGAGCGCGGCGGATATCCAGATGACCCAGTCCCCAAGCTC
Trast VL-R	ACAGGTCTCATACGTTTGATCTCGACCTTGGTACCCT
Trast VH-F	CATTCTCTGCATCTGCCGCAGACGTTCAGCTGGTGGAGTCTGGCGGTGGC
Trast VH-R	CATGGTCTCTAAGCTGAGGAGACGGTGACCAGG
Bid-F	ATCCGCCGCGCTCGC
Bid-R	GTCTGCGGCAGATGCAGAGAATG

### Phage display of a HER2-binding Fab using GBid

**Figure 3:**
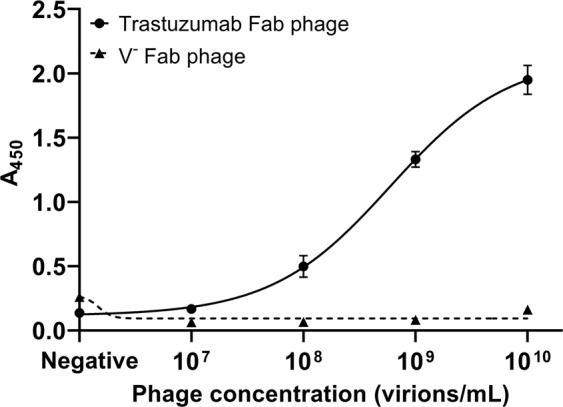
Phage display of a HER2-binding Fab using GBid. Phage displaying either Trastuzumab Fab or only the constant regions of the Fab (V^−^ Fab) were incubated in HER2-coated ELISA plates at different concentrations followed by detection of bound phage with anti-M13 antibody HRP conjugate and a colorimetric substrate. The “Negative” condition in the X-axis refers to wells coated with Dulbecco’s phosphate-buffered saline (DPBS) instead of HER2 and incubated with 10^10^ virions/mL. Values are the mean ± SD of duplicate measurements.

To demonstrate the ability of GBid to support the display functional Fabs on phage, we cloned the variable domains of the HER2-binding Fab Trastuzumab into phagemid pGBid (Fig. 1A) using the two-step GBid approach depicted in Fig. 2 to create the phagemid pGBid Trast Fab. M13 phage particles produced with SS320 *E. coli* cells carrying pGBid Trast Fab or the insert-free parental phagemid pGBid were evaluated for binding to immobilized HER2 extracellular domain^21^. As shown in Fig. 3, the Trastuzumab Fab-displaying phage exhibited dose-dependent binding to the immobilized HER2, while the phage lacking any variable domains (V^−^ Fab phage) did not bind HER2. This result indicates that the bi-directional promoter expression system contained in the pGBid phagemid is able to effectively display functional Fabs on the P3 coat protein of M13 bacteriophage.

It is noted that for this study phage was produced in the absence of IPTG induction as we observed that the leaky expression of the lac repressor-controlled BidP is adequate for production of Fab-displaying phage. Induction of Fab expression with 0.1 mM IPTG during M13K07 helper phage-assisted phage production resulted in a >100-fold reduction in the phage yield and furthermore made little difference to HER2 binding (Supplementary Table S1, Supplementary Fig. S1), indicating that induction with 0.1 mM IPTG does not significantly increase phage surface display levels of the Fab.

### Optimization of Golden Gate reaction setup

The reaction conditions for Golden Gate assembly recommended by the enzyme manufacturer New England Biolabs (NEB) are not ideal for large-scale Fab library creation. For example, 15 units (0.75 μL) of the enzyme BsaI-HFv2 and 500 units (1.25 μL) of T4 DNA ligase are recommended by NEB for a 20-μL single-fragment assembly reaction containing 50 ng of the destination vector^22^, which would necessitate 15,000 units of BsaI-HFv2 (cost > $800) and 500,000 units of T4 DNA ligase (cost > $1250) for a library setup containing 50 μg of the pGBid destination vector. Moreover, while a 1-h incubation at 37 °C yields good cloning efficiency for a one-fragment assembly^16^, temperature cycling between 37 °C (optimal for BsaI-HFv2) and 16 °C (optimal for T4 DNA ligase) was reported to yield the highest efficiencies^22,23^. We sought to define a Golden Gate reaction setup that was both cost-effective and able to access high cloning efficiencies with a minimum number of temperature shifts that could be performed manually (in water baths or heat blocks) rather than in a thermocycler which is limited to microliter volumes.

**Figure 4:**
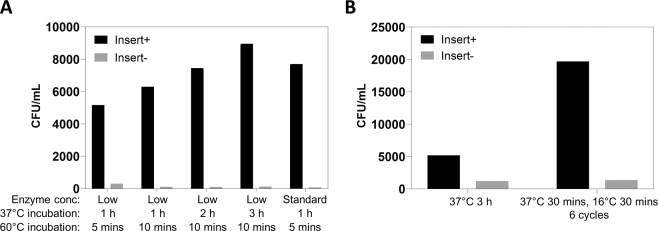
Defining Golden Gate reaction conditions for maximum scalability. The efficiency of incorporation of a VL-BidP-VH library insert derived from a hCD20-immunized chicken IgY repertoire into phagemid pGBid was monitored as colony-forming units (CFU) per mL of *E. coli* suspension following chemical transformation of 20 μL competent *E. coli* with 1 μL Golden Gate reaction mixture and recovery in 1 mL LB medium. Experimental details are provided in the “Methods” section under the subheading “Golden Gate optimization”. (**A**) Effect of reducing the concentration of the enzymes *Bsa*I-HFv2 and T4 DNA ligase to 10% (“Low”) of the manufacturer’s recommended concentration (“Standard”). Different lengths of incubation were evaluated at 37 °C and 60 °C. The rightmost bar represents the Golden Gate reaction conditions recommended by the enzyme manufacturer NEB. (**B**) Comparison of the Golden Gate assembly efficiency of a single 3-h 37 °C incubation versus 6 cycles of 37 °C 30 mins, 16 °C 30 mins. In both cases the reactions contained 10% of the manufacturer’s recommended enzyme concentration and were incubated at 60 °C for 10 minutes as the final step. Data are representative of two independent experiments.

We first investigated the impact of reducing the concentration of the enzymes in the reaction to 1.5 units BsaI-HFv2 and 50 units T4 DNA ligase per 20-μL reaction, i.e. 10% of the manufacturer’s recommended amounts, with incubation at 37 °C for 1 h, 2 h, or 3 h. We also tested the effect of changing the final 60 °C “background removal” incubation step from 5 to 10 minutes. As shown in Fig. 4A, reducing the manufacturer’s recommended enzyme concentration by 10-fold (“low” enzyme concentration) had minimal negative impact on the single-fragment Golden Gate assembly efficiency. In fact, the efficiency of the assembly with the low enzyme concentrations following a 3 h incubation at 37 °C marginally outperforms that achieved with the manufacturer’s recommended conditions using the high enzyme concentrations. The longer 10-minute incubation at 60 °C also increased the assembly efficiency consistent with previous observations^16^, a phenomenon which may be related to more complete inactivation of T4 DNA ligase^24^.

We next compared the effect of “moderate” temperature cycling between 37 °C and 16 °C – specifically 30 minutes at each temperature, 6 cycles (~6 h total reaction time) – to a single 3 h incubation at 37 °C, using the low-enzyme-concentration reaction setup (Fig. 4B). A 10-minute incubation at 60 °C was included at the end of both reactions. The temperature cycling incubation yielded ~4-fold more Golden Gate transformants than the single-temperature approach (Fig. 4B), supporting a large-scale Golden Gate Fab library creation setup in which a tube containing several mL of a reaction is moved between two water baths/heat blocks at 30-minute intervals. It is noted that the absolute number of transformants obtained for the 37 °C 3-h incubation condition in the temperature cycling study (Fig. 4B) was lower than that obtained in the first experiment (Fig. 4A), a reflection of the fact that the two experiments were conducted on different days, with the Fig. 4B study using a different batch of competent cells and *Bsa*I-HFv2 enzyme. Since experiments on a given day were set up using a common reaction master mix, comparison of data within individual charts is meaningful.

### Library construction and selection of hCD20-specific antibodies

To evaluate the ability of GBid to enable discovery of novel antibodies, a large-scale phage display Fab library was constructed from the IgY variable domains of chickens immunized with hCD20-displaying chicken HD11 macrophage-like cells according to the method depicted in Fig. 2. Overlap extension (OE) PCR of the chicken VL (chVL), BidP and chicken VH (chVH) fragments yielded a dominant chVL-BiD-chVH fragment of the correct size (1070 bp) accompanied by additional non-specific products that manifested as a smear in agarose gel electrophoresis (Supplementary Fig. S3A). It was not immediately clear whether the undesired products would be incorporated into the pGBid vector during Golden Gate cloning, so we attempted to use the crude OE PCR-generated chVL-BidP-chVH product without agarose gel purification. A 22.5-mL Golden gate reaction (2 × 11.25 mL) was performed with 56.2 μg pGBid and 18.4 μg chVL-BidP-chVH insert according to the optimized conditions determined earlier (Fig. 4) to generate a M13 GIII minor coat protein-fused chicken-human Fab library. This library was concentrated, exchanged into high-purity water, and used to electroporate SS320 *E. coli*, yielding 10^10^ transformants from ten 400-μL transformations. Colony PCR^25^ was carried out on randomly selected transformants using the flanking primers chVL-R and chVH-R to gauge the proportion of clones carrying the full-length chVL-BidP-chVH insert. Fifteen out of 19 clones (78.9%) contained the correct 1070 bp insert (Supplementary Fig. S3B), indicating that gel-purification of the 1070 bp Fab library insert is not necessary for efficient Golden Gate incorporation into pGBid.

**Figure 5:**
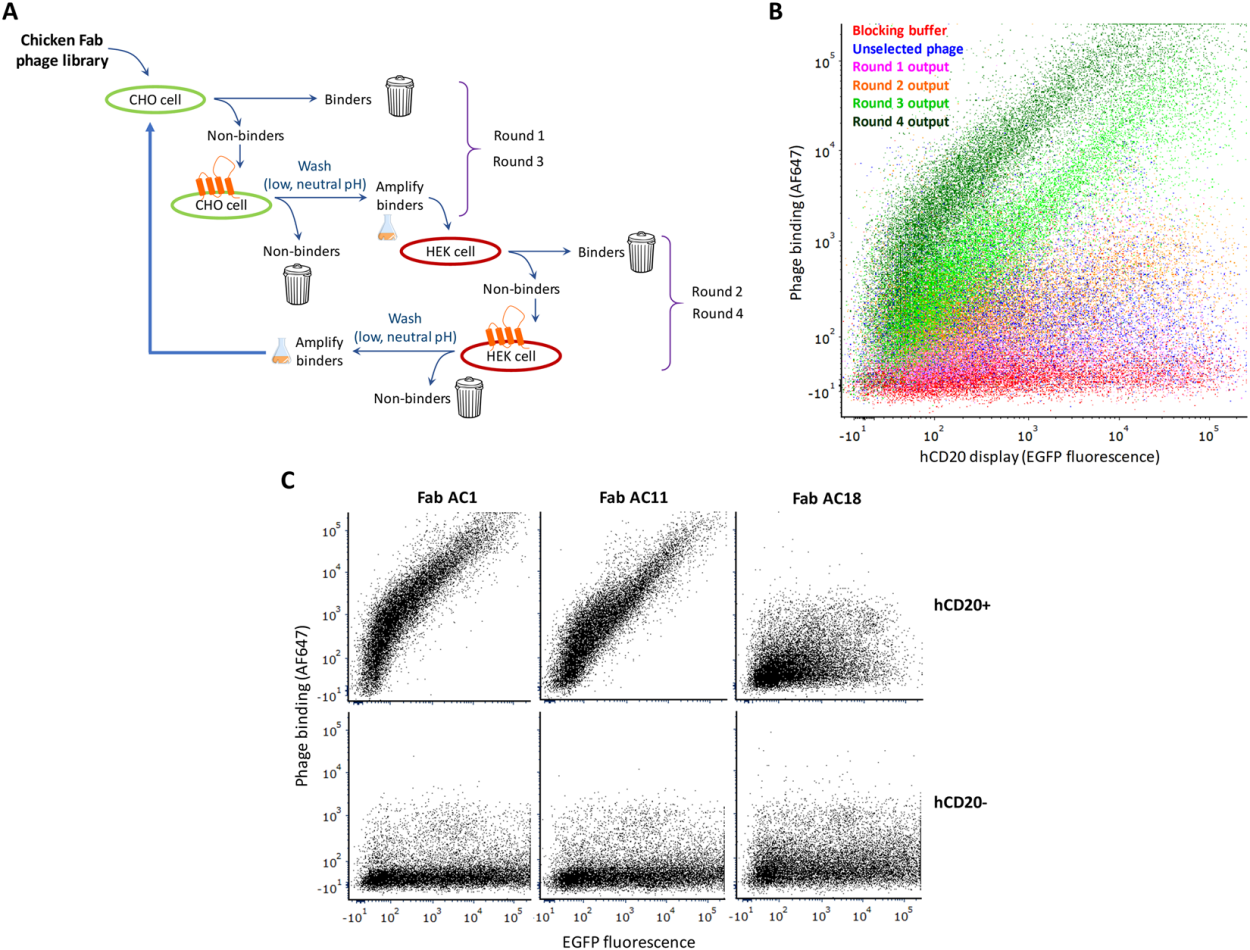
Whole cell panning for hCD20-binding chicken-human chimeric Fabs. (**A**) Overview of selection strategy. Cells transfected with hCD20 expression plasmid are diagrammatically depicted with an orange membrane protein on the cell surface while cells transfected with insert-free expression plasmid are shown without the orange surface protein. (**B**) Flow cytometric evaluation of binding of phage pools to EGFP-hCD20 CHO cells after four rounds of selection. (**C**) Evaluation of binding of three unique 4^th^-round-enriched monoclonal Fab-displaying phage to EGFP-hCD20- (upper) or EGFP-transfected (lower) CHO cells. Phage binding was detected by Alexa Fluor 647 (AF647)-labeled anti-M13 antibody.

We next set out to identify hCD20-binding Fabs within the chicken-human chimeric Fab library. SS320 *E. coli* cells transformed with the Golden Gate-created pGBid Fab library were infected with M13KO7 helper phage and the progeny phage displaying the Fab library was purified by polyethylene glycol (PEG) precipitation. The overall panning process is shown in Fig. 5A. In each round, the phage library was panned first over mock-transfected cells in a counter-selection and the non-binding phage (supernatant) from this counter-selection was subsequently panned on hCD20-transfected cells in a positive selection. After several washes with low-pH and neutral-pH wash buffers to remove weakly bound and unbound phage, phage eluted from the hCD20-transfected cells were amplified in SS320 *E. coli* and used to perform a subsequent round of counter-selection and positive selection. CHO-K1 cells were used in selection rounds 1 and 3, while HEK293T cells were used in rounds 2 and 4. The counter-selection on mock-transfected cells serves to deplete the phage pools of clones that non-specifically bind non-hCD20 cell-surface factors, while the alternation between the CHO-K1 and HEK293T cells helps minimize the amplification of cell-specific phage clones that escape the counter-selection.

It is noted that after two rounds of counter-selection/selection, most of the selected phagemids were observed to lack the full-length chVL-BidP-chVH insert (Supplementary Material; Fig. S4). To recover/enrich the full-length Fabs, the small remaining fraction of full-length chVL-BidP-chVH insert was selectively extracted via agarose gel purification, amplified by PCR, and re-cloned into pGBid using the GBid approach (Supplementary Material; Fig. S5). Phage produced from the re-cloned phagemid pool was used in the subsequent round of biopanning.

To simplify the analysis of the ability of phage from different selection rounds to bind hCD20, a fusion protein, EGFP-hCD20, was constructed, allowing the cell-surface display level of hCD20 in transfected cells to be gauged via enhanced green fluorescent protein (EGFP) fluorescence intensity. The output phage pools from the four rounds of selection/counter-selection were flow cytometrically evaluated for binding to EGFP-hCD20-transfected CHO cells, indicating a progressive enrichment of phage that bind the transfected cells in a hCD20-display-dependent manner (Fig. 5B). Colony PCR and sequencing of 20 randomly selected monoclonal phagemids from selection round 4 revealed that a single full-length clone, Fab AC1, dominated the output pool (Supplementary Material). Colony PCR and sequencing of at least 20 randomly selected 3^rd^-round monoclonal phagemids revealed that even though most (85%) contained full-length (~1.1 kb) Fab inserts, the majority (56%) of 3^rd^-round VH chains contained nucleotide insertions/deletions that create frameshifts in the open reading frame or mutations that introduce premature stop codons (Supplementary Material). Of the 18 out of 46 3^rd^-round monoclonal phagemids containing fully-expressed Fabs, 5 exhibited hCD20-specificity and all 5 resembled or were identical to Fab AC1 (Supplementary Material).

**Figure 6:**
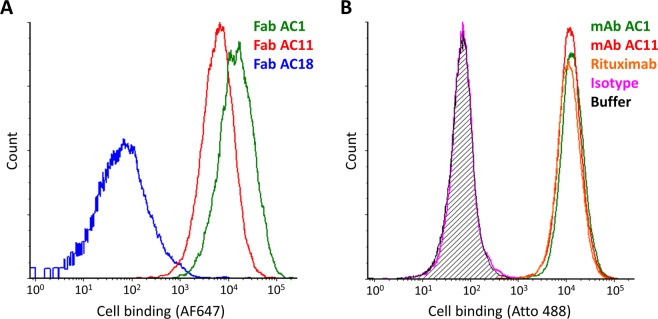
Evaluation of binding of monoclonal Fab-displaying phage and reformatted full-length antibodies to a B lymphoma cell line natively expressing hCD20. (**A**) Phage displaying three monoclonal Fabs – AC1, AC11 and AC18 – from the 4^th^ round of biopanning were incubated with Raji cells prior to staining with AF647-labeled anti-M13 antibody and flow cytometric analysis for binding. Fabs AC1 and AC11 but not Fab AC18 bind hCD20-positive Raji cells, consistent with the Fab phage binding study in hCD20-transfected CHO cells (Fig. 5C). (**B**) The full-length chimeric chicken-human mAb versions of two of the unique hCD20-binding Fabs enriched in the 4^th^ round of whole-cell phage panning, AC1 and AC11, a positive-control hCD20-binding antibody Rituximab, and an isotype control antibody Trastuzumab, were labeled with Atto 488 and 5 μg/mL mAbs or staining buffer were incubated with Raji cells prior to flow cytometric analysis. The histogram for the staining-buffer treated cells is filled with black stripes.

To identify rare non-Fab AC1 hCD20-specific clones within the 4^th^-round phagemid pool, the dominant clone Fab AC1 was selectively depleted by treatment with restriction enzymes that specifically cleave within its VH complementarity-determining regions (CDRs) (Supplementary Material), revealing the presence of a second completely unique hCD20-specific clone, Fab AC11. Phage displaying Fabs AC1 and AC11 bind both EGFP-hCD20-transfected CHO cells in a hCD20 display-dependent manner (Fig. 5C), indicating specific interaction with hCD20. The same two monoclonal phage bind Raji cells which natively express hCD20 (Fig. 6A). A third phage clone selected for comparison, Fab AC18, does not bind either the transfected CHO (Fig. 5C) or Raji cells (Fig. 6A).

Next, Fabs AC1 and AC11 were converted to full-length chimeric chicken-human mAbs for flow cytometric evaluation of hCD20 binding. As shown in Fig. 6B, both mAbs AC1 and AC11 bind Raji cells comparably to an in-house version of the clinically used anti-hCD20 antibody, Rituximab, while the isotype control antibody does not bind the same cells (Fig. 6B), indicating that the paratopes of both Fabs faithfully recapitulate the binding mode of the full-length mAbs. Studies are ongoing in our lab to further characterize the anti-hCD20 activity of these chicken-human chimeric anti-hCD20 mAbs.

## Discussion

Of the two main phage display antibody formats for antibody discovery/engineering – Fabs and scFvs, Fabs are considered more “IgG-like” than scFvs due to the presence of constant domains and are therefore the more favored format for full-length IgG discovery. As a corollary to this proposition, it has been observed that the target-binding activity of scFvs can decrease dramatically or even disappear altogether upon reformatting to full-length IgGs^5,7^. Unfortunately, Fab display library construction tends to be more labor-intensive relative to scFvs and often requires PCR amplification of the human constant domains, thus risking the introduction of unwanted mutations into these regions. In this study we describe the development and application of a simple, scalable approach – Golden Gate assembly with a bi-directional promoter (GBid) – for phage display Fab library creation that greatly simplifies the process, making it essentially equivalent to or easier than the scFv library creation process in cloning complexity. In GBid, the antibody constant domains are incorporated into the phagemid vector pGBid adjacent to recognition sites for a Type IIS restriction enzyme, and the library insert is composed of only the antibody variable domains and a central bi-directional promoter (BidP), thus allowing seamless one-pot cloning without the need to PCR-amplify the constant domains.

The GBid concept relies on a bi-directional promoter, BidP, to direct the display of functional Fabs on the phage coat. Apart from one study in which an overlapping bi-directional promoter was used to increase long-term promoter stability in a synthetic gene circuit application^26^, to our knowledge, this is the first report of a prokaryotic bi-directional promoter designed specifically for simultaneous expression of two proteins. The expression of the heavy and light chains of the Fabs from separate promoters as opposed to from a single promoter containing two ribosome-binding sites ensures robust expression of both chains^14^. BidP comprises a constitutively repressed *tac* promoter/*lac* operator system^17^ that allows just enough leaky expression of the Fab light and heavy chains for efficient display of functional Fabs on the phage coat. While this promoter is inducible by IPTG, we found that the induction with IPTG at concentrations as low as 0.1 mM impaired phage production yields by >100-fold and furthermore had little impact on the phage display of functional Fab (Supplementary Table S1, Supplementary Fig. S1). The significant drop in phage yields following IPTG induction may be a consequence of an oversized metabolic burden on the host *E. coli* cells that derives from the unusually high strength of the engineered P*tac* promoter^17^. The other major components of the BidP cassette are the flanking DsbA signal sequences which direct expression of the VH and VL chains to the cotranslational SRP pathway. The DsbA signal sequence was chosen over more conventional phage display signal peptides (eg. ompA, pelB) which utilize the Sec post-translational translocation pathway because it has been reported to support the efficient periplasmic translocation and phage display of a wide range of proteins, particularly proteins that tend to fold rapidly and/or adopt a rigid structure^18^.

To enable incorporation of library inserts without the introduction of unwanted amino acids at junctions, the widely used Type IIS restriction enzyme *Bsa*I was chosen for cloning. The use of Type IIS enzymes for seamless cloning of DNA fragments has recently gained in popularity due to the different location of the enzyme recognition site relative to the cut site^27^. Type IIS enzymes have traditionally been used for Golden Gate assembly of multiple fragments into a destination vector^23,27–32^ and, except for a few examples of lower-efficiency multi-fragment library assemblies^15,33,34^, have not been used for the construction of large diverse libraries. This reality is surprising, given the overall simplicity and seamless integration of insert with vector that Golden Gate affords. We suspect that the lack of use of the Golden Gate method for library construction may derive from the reported requirement of a high concentration of expensive enzymes (Type IIS restriction enzyme, T4 DNA ligase) and the apparent need for a large number of temperature shifts between the optimum temperatures for the restriction enzyme (typically 37 °C) and ligase (16 °C) to achieve a high insert-to-vector incorporation efficiency^22^. While the temperature shifting paradigm is convenient for microliter-scale reaction volumes, reactions for large libraries are typically in the milliliter scale and frequent temperature shifts can be impractical. In this study, we define conditions for achieving a high insert-to-vector incorporation efficiency that do not require prohibitively high enzyme concentrations and utilize only a small number of temperature shifts. Specifically, we demonstrate effective Golden Gate assembly of a single VL-BidP-VH fragment into the pGBid phagemid vector using only 1.5 units of BsaI-HFv2 and 50 units of T4 DNA ligase per 50 ng of vector, 10-fold lower amounts than that recommended by the enzyme manufacturer^22^, as well as only 6 cycles of incubation at 37 °C for ~30 minutes followed by 16 °C for ~30 minutes.

As discussed above, a compelling application of Golden Gate cloning is the assembly of several fragments into a destination vector. Simultaneous assembly of several gene fragments, however, comes at the cost of a lower overall recombination efficiency^22,23^, an undesirable trait in applications where the desired output is a large diverse library as opposed to a single recombinant construct. An important element of the GBid design is the need to incorporate only one fragment into the destination vector for library creation, thus allowing libraries of maximum size/diversity to be created. Although the three separate fragments for incorporation into the pGBid phagemid vector – VL, BidP, and VH – could potentially have been designed with their own Type IIS restriction enzyme recognition sites for a multi-fragment Golden Gate assembly, we chose not to go this route in the interests of maximizing library output. Instead, the three fragments are first combined via a 3-fragment overlap extension PCR reaction and the recombined fragment is utilized for a single-fragment Golden Gate assembly reaction.

Given that GBid involves only one insert fragment, the classical cloning approach of digestion of the destination vector and insert with uniquely cutting restriction enzymes followed by a separate ligation reaction might seem like an equally appropriate method for library construction. This is certainly a viable approach for non-Type IIS restriction sites and has of course long been the go-to method for library construction. For the Type IIS restriction enzyme *Bsa*I, however, we observed that the classical digestion-followed-by-ligation approach is significantly less efficient at producing recombinant phagemid, with only ~20% of transformants of the ligation reaction containing the correctly cloned insert. Lower recombination efficiency with separate Type IIS enzyme digestion and ligation reactions relative to a one-pot restriction-ligation has also been observed by others^15^. We therefore believe that the one-pot approach is not only attractive for its reduced number of DNA manipulations and convenience, but actually may be necessary to maximize Fab library output while exploiting the seamless integration afforded by Type IIS restriction enzymes.

The application of GBid for antibody discovery was demonstrated by the identification of two unique antibodies against the complex multi-transmembrane protein hCD20 from the IgY variable domains of immunized chickens. Multi-transmembrane or multi-pass membrane proteins represent an important but extremely challenging class of targets for antibody discovery. The difficulties associated with identifying binders to these targets stem primarily from dependence on a lipid membrane for expression and therefore general inability to be purified to high degree for immunization and biopanning. Various formats have been used to present complex membrane-dependent antigens for antibody discovery^35^. Of these, display on whole cells offers the most native-like conformation but also presents some of the most daunting challenges for biopanning given the high degree of background cell-surface “noise” that threatens to drown out target-specific binding events^36^. The GBid approach fostered success in this challenging endeavor by enabling the facile creation of a large library of 10^10^ potentially unique transformants containing ~80% full-length Fab inserts, approaching the maximum phage display library size empirically achieved using “traditional” library creation approaches^37^. It is duly noted that our ability to create this large library was a function not only of the effective GBid method, but also the efficiency of our electrocompetent cells.

In addition to the use of GBid, several other steps were taken to help ensure success in our quest to discover anti-hCD20 antibodies: (*i*) Use of an immune library enriched for hCD20-binders, as opposed to a naïve or completely synthetic library. Indeed, the discovery of binders to multi-pass membrane proteins from naïve libraries has reportedly proven difficult/impossible^36^. The choice of chickens as a source of immune diversity was particularly appealing given the large phylogenetic distance of chickens from humans, allowing the discovery of binders to potentially important conserved mammalian epitopes that would not be accessible in more conventional immune hosts such as mice. In addition, chicken immunoglobulins comprise single VH and VL genes, a feature that further simplifies library construction as only one set of oligonucleotide primers is needed for cloning of each antibody chain^38^. On the strength of these attractive features, the use of chicken immune repertoires for discovery of monoclonal antibodies has recently gained in popularity^39–42^. (*ii*) The performance of selections in a dual counter-selection/positive selection format that first depletes phage pools of background cell-surface binders on mock-transfected cells prior to incubation with hCD20-transfected cells. (*iii*) The use of alternating cell lines – CHO-K1 and HEK293T – in different rounds of selection to minimize the likelihood of enriching binders to cell-specific factors. Panning on alternating cell lines has been previously used for the identification of antibodies against targets displayed on whole cells^36,43^. (*iv*) The application of low-pH wash steps during panning to remove phage that adhere to cells through weak non-specific interactions. This approach was previously employed during whole-cell phage panning campaigns to minimize the enrichment of insert-free phage clones^36,44^. Even with the stringent washing, however, a predominance of phage clones with shortened/no inserts was observed in the 2^nd^-round enriched pool, and gel-purification/amplification of the small fraction of remaining full-length chVL-BidP-chVH inserts followed by re-cloning into pGBid was therefore performed to recover/enrich Fab-displaying phage prior to the 3^rd^-round of biopanning (Supplementary Material).

It is noted that the GBid experimental setup in this study utilizes the Type IIS enzyme *Bsa*I for cloning. In order to ensure that GBid maximally represents a library’s diversity on the surface of phage, it is important that the recognition site for the Type IIS restriction enzyme used for the cloning is not commonly present within the library. In the case of the chicken immune repertoire, we did not observe any *Bsa*I sites within the non-variable DNA sequence frameworks, but it is conceivable that *Bsa*I sites might be more common within repertoires from other species. In such cases, recognition sites for other Type IIS restriction enzymes (eg. *Bbs*I, *Sap*I) can be engineered into the pGBid phagemid to replace the existing *Bsa*I sites.

In summary, we have developed a simple and scalable method – GBid – for large-scale phage display Fab library creation comprising two simple steps: a three-fragment overlap PCR and a one-fragment Golden Gate assembly. GBid-mediated Fab library creation requires very few sample manipulation steps relative to a more conventional digestion-followed-by-ligation approach, therefore minimizing the loss of valuable DNA sample. We used GBid to create a high-quality phage display library from the antibody repertoire of immunized chickens. Selection/counter-selection of the Fab library on transfected whole mammalian cells yielded two monoclonal antibodies specific to the complex multi-pass membrane protein target hCD20. We anticipate that GBid will help accelerate the creation of phage-displayed Fab libraries and the discovery of Fabs/IgGs with relevant binding activity.

## Methods

### Reagents, kits and cells

Unless otherwise specified, all oligonucleotides were purchased from Integrated DNA Technologies, enzymes and reaction buffers from NEB, and chemicals from Sigma-Aldrich. Agarose gel extractions were performed with the Zymoclean Gel DNA Recovery Kit (Zymo Research) and cleanup of PCR reactions were performed with the QIAquick PCR Purification Kit (Qiagen). SS320 *E. coli* cells were obtained from Lucigen and made electrocompetent for transformation by washing with cold high-purity water as described in the NEB website^45^ and elsewhere^37^. M13KO7 helper phage was propagated in XL1-Blue *E. coli* cells from an original stock purchased from NEB (catalog # N0315S), and a concentrated working stock made as described earlier^37^. HEK293T cells and CHO-K1 cells were cultured from lab stocks in DMEM medium supplemented with 10% fetal bovine serum. Expi293F cells were from ThermoFisher (catalog # A14527) and cultured in Expi293 Expression Medium according to the manufacturer’s recommendations. Raji cells were purchased from American Type Culture Collection (ATCC, catalog # CCL-86) and cultured in RPMI 1640 supplemented with 10% fetal bovine serum.

### Construction of phagemid pGBid

A CLκ-BidP-CH1 gene fragment containing (1) flanking *Kas*I and *Sal*I restriction sites, (2) *E. coli* codon-optimized genes for CLκ and CH1 positioned in opposing directions and (3) a central BidP sequence (Fig. 1B) was synthesized by Integrated DNA Technologies. This fragment was digested with *Kas*I and *Sal*I and cloned upstream of the M13 GIII minor coat protein in a P3 display phagemid^19^ to create pGBid.

### Creation of phage displaying a HER2-binding Fab

Expression vectors containing the heavy and light chains of the HER2-binding mAb Trastuzumab were a kind gift of Dr. Sally Ward and have been reported previously^46^. The Trastuzumab VL domain was amplified from the light chain plasmid using the primers Trast VL-F and Trast VL-R and VH was amplified from the heavy chain plasmid using the primers Trast VH-F and Trast VH-R. BidP was amplified from pGBid using the primers Bid-F and Bid-R. The sequences of all primers are in Table 1. The VL, BidP and VH fragments were agarose gel-purified and 10 ng of each of the three fragments were used in a 20-μL primer-free OE assembly reaction containing 1X Phusion HF Buffer, 0.2 mM dNTPs, and 0.2 units Phusion DNA Polymerase. Primer-free OE was performed as follows: 98 °C 30 s, followed by 10 cycles of 98 °C 10 s, 50 °C 30 s, 72 °C 30 s, followed by 72 °C 5 mins. At the completion of this reaction, 0.1 μL fresh Phusion DNA Polymerase was added and another 10 cycles of overlap extension performed. Four μL of this primer-free OE reaction were used as template in an amplification reaction containing 1X Phusion HF Buffer, 0.2 mM dNTPs, 0.5 μM of primers Trast VL-R and Trast VH-R, and 1 unit Phusion DNA Polymerase. Thermocycling was carried out as follows: 98 °C 30 s, followed by 8 cycles of 98 °C 10 s, 72 °C 45 s (no separate lower-temperature annealing), followed by 72 °C 5 mins. The completed reaction was cleaned up using the QIAquick PCR Purification Kit (Qiagen). The concentration of the dominant 1 kb band corresponding to the VL-BidP-VH fragment was quantified by band-intensity comparison with the Quick-Load Purple 1 kb Plus DNA Ladder (NEB). Golden Gate-mediated incorporation into pGBid was performed using 50 ng of destination vector pGBid and a 2:1 insert:pGBid molar ratio in a 20-μL reaction incubated at 37 °C for 1 h followed by 60 °C for 5 minutes according to the recommendations of the enzyme manufacturer NEB^22^. The completed Golden Gate reactions were diluted 10-fold in sterile high-purity water and 1 μL was used to transform SS320 *E. coli* cells. The construct containing the Trastuzumab Fab insert was named pGBid Trast Fab.

To confirm the display of Trastuzumab Fab, phage particles containing pGBid Trast Fab and the insert-free pGBid vector (negative control) were produced and concentrated following standard protocol^13^. Titers of infectious phage particles were determined by measuring optical density at 268 nm^37^ and confirmed by colony enumeration following infection of SS320 *E. coli* with serially diluted phage and plating on carbenicillin LB agar plates^47^. Titers of infectious phage particles produced in the presence and absence of IPTG are provided in Supplementary Table S1.

### Phage ELISA to evaluate HER2 binding

The soluble extracellular domain of HER2 (ErbB2) was a gift from Prof. Timothy Adams of the Commonwealth Scientific and Industrial Research Organisation (CSIRO)^21^. Nunc MaxiSorp uncoated ELISA plates (BioLegend) were coated overnight at 4 °C with 100 μL of either ErbB2 (2 μg/mL in DPBS) or DPBS. The coating solution was removed and the wells blocked with 300 μL blocking buffer (DPBS with 0.05% Tween 20 and 2% bovine serum albumin [BSA]) at ambient temperature for 2 h. The blocking buffer was removed, the wells washed once with 300 μL washing buffer (DPBS with 0.05% Tween 20), and 100 μL of phage at the indicated concentrations in binding buffer (DPBS with 0.05% Tween 20, 0.5% BSA) were incubated in the wells for 1 h at ambient temperature. As a negative control, phage at the highest concentration (10^10^ virions/mL) was added to wells that were pre-treated with DPBS instead of HER2. After incubation, the wells were washed 6 times with 200 μL washing buffer and then incubated with 100 μL mouse anti-M13 antibody HRP conjugate (GE Healthcare, catalog # 27-9421-01) at ambient temperature for 1 h. The antibody solution was removed and the wells washed 6 times with 200 μL washing buffer. Bound antibody was detected by incubation with 100 μL BioFx TMB HRP substrate (Surmodics) for approximately 2 minutes to allow color development. The reaction was stopped by addition of 100 μL 1 M sulfuric acid and absorbance was measured at 450 nm using a Tecan Infinite M200 plate reader.

### Golden Gate optimization

All Golden Gate reactions were setup in a 20-μL final volume and contained 50 ng phagemid pGBid, and 1X T4 DNA ligase Buffer. “Insert+” reactions contained 16.3 ng chicken anti-hCD20 library chVL-BidP-chVH insert (see “Library construction and selection of hCD20-specific antibodies” section under Results) and “Insert−” reactions contained water in its place. According to the recommendations of the manufacturer NEB, the “standard” setup contained 15 units BsaI-HFv2 and 500 units T4 DNA ligase^22^. The “low enzyme concentration” setups contained 1.5 units BsaI-HFv2 and 50 units T4 DNA ligase. Incubations were performed in a Bio-Rad C1000 Touch Thermal Cycler. One μL of the completed reactions was used to transform 20 μL high-efficiency competent NEB 5-alpha *E. coli* cells (NEB) per the manufacturer’s recommended protocol, the cells were recovered in 1 mL LB medium at 37 °C for 45 minutes, and 10 μL or 70 μL was plated on LB agar plates containing 50 μg/mL carbenicillin prior to overnight incubation at 37 °C and colony enumeration. Plots indicate the number of colonies expected from an entire transformation, corresponding to 1 mL of transformed cells.

### Library construction and selection of hCD20-specific antibodies

The creation of hCD20-displaying chicken macrophage-like cells, immunization of chickens with these cells, evaluation of reactivity of immunized chicken serum for hCD20-positive cells, generation of first-strand cDNA from the spleen and bone marrow of immunized chickens, and amplification of the chVL and chVH library fragments using the primer pairs chVL-F/chVL-R and chVH-F/chVH-R (Table 1) are described in the Supplementary Material section. Overlap extension PCR of the chVL, BidP and chVH fragments and subsequent PCR cleanup was performed as described above under “Creation of phage displaying a HER2-binding Fab”, except that the primer-free OE reactions were linearly scaled up to a 50-μL volume each containing 50 ng VL, 65 ng VH, and 50 ng BidP fragments, and 20 of these reactions were performed. Also, 23 μL of the primer-free OE reactions were used as a template for each subsequent 50-μL amplification reaction (40 reactions total) utilizing 0.5 μM of the flanking primers chVL-R and chVH-R.

Two 11.25-mL Golden Gate assembly reactions were set up in 15-mL conical tubes, each containing 28.1 μg phagemid pGBid, 9.2 μg chVL-BidP-chVH fragment (2:1 insert:vector molar ratio), 1X T4 DNA ligase buffer, 844 units BsaI-HFv2, and 28,125 units T4 DNA ligase. The tubes were manually immersed in water baths set to 37 °C or 16 °C as follows: 6 cycles of (30–35 minutes at 37 °C, 30–35 minutes at 16 °C) followed by 60 °C for 15 minutes. The completed reactions within each tube were pre-concentrated to a final volume of 0.75–0.90 mL using 10 K MWCO Amicon Ultra-15 centrifugal filter units (EMD Millipore). The pre-concentrated samples were further concentrated using ethanol precipitation to a final combined volume of 300 μL sterile reverse-osmosis water as follows: 1/10^th^ volume of 3 M sodium acetate pH 5.5 was added to the samples followed by 2.33 volumes of 100% ethanol and the samples were incubated at −20 °C for >12 h. The samples were distributed into 6 microcentrifuge tubes and centrifuged at 18,000 *g* and 4 °C for 20 minutes. The supernatants were removed and the DNA pellets washed twice with 500 μL 75% ethanol with centrifugation at maximum speed 4 °C for 10 minutes for each wash step. The DNA pellets were air-dried at ambient temperature for 5–10 minutes after complete removal of ethanol and the pellets solubilized in a final combined volume of 300 μL sterile reverse osmosis water. The DNA sample was stored at −20 °C.

To generate the Fab-displaying phage library, two batches of 5 electroporations of electrocompetent SS320 *E. coli*^37^ were performed, using 380 μL electrocompetent cells and 20 μL purified/concentrated Golden Gate reaction per electroporation. Chilled 2-mm gap cuvettes (Fisher Scientific) and a setting of 2500 V was used on a BTX ECM 399 electroporation system. The cells from 5 electroporations were combined into 100 mL prewarmed SOC medium and incubated at 37 °C with shaking at 240 rpm for 15 minutes. Thereafter, carbenicillin was dispensed to a final concentration of 50 μg/mL and incubation at 37 °C and 240 rpm was continued for an additional 30 minutes. A small amount of these cells (for each batch of 5 electroporations) was serially diluted and plated on 50 μg/mL carbenicillin-containing LB agar plates for determination of library size, indicating a combined representation of 1 × 10^10^ independent transformants. After recovery, both batches of electroporated cells were combined (~200 mL total) and added to a 2.8-L baffled Erlenmeyer flask containing 900 mL prewarmed 2xYT medium supplemented with 5 μg/mL tetracycline and 50 μg/ml carbenicillin and the cells incubated at 37 °C and 200 rpm until the optical density at 600 nm (OD_600_) reached ~2 (approximately 8 h). 25 mL of the OD_600_ ~2 culture was used to inoculate 500 mL of 2xYT medium supplemented with 5 μg/mL tetracycline and 50 μg/mL carbenicillin and the culture incubated at 37 °C and 240 rpm until OD_600_ ~0.5 (approximately 2 h). The remainder of the OD_600_ ~2 culture was concentrated 40-fold and cryopreserved in 1-mL aliquots for later production of library phage as required. Upon reaching OD_600_ ~0.5, the 500-mL culture was inoculated with M13KO7 helper phage to a final concentration of 10^10^ pfu/mL and the cells incubated at 37 °C without shaking for 20 minutes and then with shaking at 240 rpm for 30 minutes. Kanamycin (50 μg/mL) was added and the culture grown for an additional 15 h at 30 °C. Phage was harvested from the supernatant by PEG/NaCl precipitation^37^, resuspended in 1 mL DPBS, titered by OD_268_ measurement^37^, and stored at −80 °C in aliquots.

For whole-cell panning, CHO-K1 cells or HEK293T cells were seeded in 150-mm tissue culture dishes at 7.9 × 10^6^ and 2 × 10^7^ cells per dish, respectively. Approximately 20 h after seeding, the cells were transfected with 16 μg (CHO) or 8 μg (HEK293T) plasmid DNA using jetPRIME (Polyplus-transfection) reagent per the manufacturer’s recommended protocol at a 2:1 jetPRIME:DNA (μL:μg) ratio. Mock transfections utilized pCMV5 plasmid^48^ and hCD20 transfections used pCMV5-hCD20 in which the cDNA for hCD20 (MS4A1) was cloned into the *Cla*I and *Bam*HI sites downstream of the CMV promoter. Approximately 48 h post transfection, cells were detached either by a DPBS wash followed by a 5-minute incubation with 1X citric saline solution (135 mM potassium chloride, 15 mM sodium citrate) at 37 °C (CHO) or vigorous pipetting with DPBS (HEK293T). Cells were washed with DPBS and resuspended with phage blocking buffer (DPBS/10% FBS) to a density of >1 × 10^7^ cells/mL (CHO) or >2 × 10^7^ cells/mL (HEK293T). One mL of the mock- and hCD20-transfected cell suspensions were transferred into sterile siliconized 2-mL microcentrifuge tubes and incubated at 4 °C with tumbling for ≥30 minutes. At the same time, the input phage was diluted in phage blocking buffer to 10^13^ phage/mL (unselected initial library phage pool) or 10^12^ phage/mL (selected/amplified phage from the previous round of selection) in 2-mL siliconized microcentrifuge tubes that had been pre-blocked with phage blocking buffer and tumbled at 4 °C for ≥30 minutes. The mock-transfected cells were centrifuged at 370 g for 3 minutes at 4 °C and the blocking supernatant removed and replaced with 1 mL of the blocked input phage. The mixture was tumbled at 4 °C for ≥2 h, pelleted by centrifugation and the supernatant used to resuspend the cell pellet of the blocked hCD20-transfected cells. The hCD20-transfected cells/phage mixture was incubated at 4 °C with tumbling for ≥2 h. The mixture was pelleted and washed 3 times with 1 mL cold low-pH phage wash buffer (DPBS/5% FBS, pH 5.0 adjusted with citric acid) with each wash involving tumbling at 4 °C for 5 minutes and centrifugation at 370 g and 4 °C for 3 minutes. The cells were washed an additional 4 times with cold phage wash buffer (DPBS/5% FBS). Cell-bound phage were eluted by incubation with 0.5 mL glycine elution buffer (100 mM glycine, 0.1% BSA, 0.5 M NaCl, pH 2.2) at ambient temperature with tumbling for 10 minutes. Cells were pelleted at 370 g for 3 minutes and the supernatants transferred to clean siliconized microcentrifuge tube that had been previously blocked with phage blocking buffer. The sample was pelleted once more at 10,000 g for 30 seconds to remove residual debris and transferred to a clean pre-blocked siliconized tube. 15.5 μL of 2 M Tris was added to neutralize the pH and the phage used to infect 20 mL (panning round 1) or 5 mL (rounds 2, 3 and 4) mid-log phase (OD_600_ 0.5–0.8) SS320 *E. coli* cells. Infection was carried out at 37 °C without shaking for 20 minutes then with shaking at 240 rpm for 30 mins. Cultures were then diluted 10-fold in 2xYT medium supplemented with 5 μg/mL tetracycline and 50 μg/mL carbenicillin and grown at 37 °C and 240 rpm to mid-log phase (OD_600_ 0.5–0.8). M13KO7 helper phage was added to a final concentration of 10^10^ pfu/mL followed by incubation at 37 °C without shaking for 20 minutes and then with shaking at 240 rpm for 30 minutes. Kanamycin (50 μg/mL) was then added and the culture incubated at 30 °C and 240 rpm overnight (15–20 h). The phage from the supernatant was precipitated with PEG/NaCl^37^, resuspended in 0.25–0.50 mL DPBS, titered by measurement of OD at 268 nm^37^, and stored at −80 °C in aliquots.

### Flow cytometric analysis of phage pools and clones

Purified phage pools from the unselected initial phage library and the outputs of selection Rounds 1–4, and monoclonal phage from Rounds 3 and 4, were tested for binding to transfected CHO cells or wild-type Raji cells using flow cytometry. CHO cells were transfected as described above with either pTRIP-EGFP hCD20 containing hCD20 with an EGFP gene fused at its N terminus under the control of a CMV promoter or pTRIP-EGFP (mock) containing just EGFP, and used two days post-transfection. Raji cells were used directly from passaging culture. Cells were resuspended in phage blocking buffer (DPBS/10% FBS) to a density of ~10^6^ cells/mL and tumbled at 4 °C for ≥30 minutes. Phage pools (8 × 10^11^ phage/mL) were also blocked in cold phage blocking buffer at 4 °C for ≥30 minutes. ~2 × 10^5^ cells were incubated with 50 μL of either cold phage blocking buffer or blocked phage in a V-bottom 96-well plate for 1 h at 4 °C with rocking. The cells were washed twice with 200 μL cold staining buffer (DPBS/1% BSA) with pelleting at 400 g (CHO) or 500 g (Raji) for 3 minutes at 4 °C, then incubated with 50 μL of 5 μg/mL anti-M13 gp8 antibody (RL-ph2; ThermoFisher, catalog # MA106604) labeled with Alexa Fluor 647 NHS ester (ThermoFisher) at 4 °C for 1 h with rocking. Fluorescent labeling of the primary antibody with Alexa Fluor 647 NHS ester was performed as described for Atto 488 NHS ester labeling of purified mAbs in the “Construction, expression and testing of full-length mAbs” section. The cells stained with the fluorescently labeled antibody were washed three times with 200 μL cold staining buffer, then analyzed on a BD LSRFortessa X-20 flow cytometer. Purified monoclonal phage for analyses were prepared from individual phagemid-containing colonies as described above for a HER2-binding Fab-displaying phage.

### Construction, expression and testing of full-length mAbs

The genes for the VL and VH domains from monoclonal Fab phagemids pGBid Fab AC1 and pGBid Fab AC11 were separately PCR-amplified and inserted downstream of the CMV promoter and upstream of the sequences for human CLκ and the human IgG1 CH1-CH2-CH3 fragment, respectively, in in-house pCOriP expression vectors derived from pCMV5^48^ containing a shortened Epstein Barr virus origin of replication (sOriP)^49^. Gene fragments encoding the VL and VH domains of Rituximab (PDB code 2OSL) were purchased from Gene Universal (Newark, DE) and cloned into pCOriP vectors in the same way. The pCOriP-VH and pCOriP-VL plasmids were used to co-transfect Expi293F suspension cells with pCOriP-EBNA1t which expresses a truncated Epstein Barr Virus Nuclear Antigen-1 (EBNA1)^50^, at a 1:1.3:0.6 weight ratio using FectoPRO transfection reagent (Polyplus-transfection). Transfection parameters of 0.35 μg DNA per mL culture and a 1:0.875 FectoPRO (μL):DNA (μg) ratio were used. Cultures were incubated at 37 °C/8% CO_2_ in plain-bottom flasks with shaking at 125 rpm in a 19-mm orbit shaker. Supernatant containing full-length mAbs were harvested 5–6 days post transfection and subjected to gravity-flow purification in Pierce disposable 5-mL plastic columns (ThermoFisher) using MabAffinity rProtein A High Flow Beads (ACROBiosystems) as described^51^. Protein A-bound mAbs were eluted 5 times with 2 mL 100 mM citrate buffer pH 3.6^50^, with each fraction being immediately neutralized with 0.39 mL 1 M Tris. Eluted fractions were pooled and concentrated to a final volume of 0.5–1 mL using 30 kDa MWCO Amicon Ultra-15 centrifugal units (EMD Millipore), exchanged into DPBS using 2-mL Zeba spin desalting columns (ThermoFisher) according to the manufacturer’s directions, flash-frozen in liquid nitrogen in aliquots, and stored at −80 °C. Protein purity was determined by SDS-PAGE and concentration measured using the Pierce BCA Protein Assay Kit (ThermoFisher). For flow cytometry experiments mAbs were fluorescently labeled as follows: Atto 488 NHS ester (Sigma-Aldrich, catalog # 41698) was dissolved in DMSO to 10 mg/mL and 2 μL of the dye was rapidly combined with 50 μg of purified mAb diluted in PBS adjusted to pH 8.2 (250 μL final volume) and the reaction was tumbled at 4 °C in the dark overnight. The labeled mAbs were exchanged into DPBS using 2-mL 7 K MWCO Zeba Spin Desalting Columns (ThermoFisher).

Raji cells were resuspended in cold phage blocking buffer to 1.5 × 10^6^ cells/mL and tumbled at 4 °C for ≥30 minutes. ~3 × 10^6^ Raji cells in a V-bottom 96-well plate were incubated with 50 μL of 10 μg/mL Atto 488-labeled mAbs in staining buffer with rocking at 4 °C for ≥1 h. Cells were washed three times with 200 μL cold staining buffer with pelleting at 500 g for 3 minutes at 4 °C and analyzed via flow cytometry as described above for the phage binding analysis.

## Supplementary information

Supplementary information
