# Supplementary material for: Golden Gate assembly with a bi-directional promoter (GBid): A simple, scalable method for phage display Fab library creation
Source: Sci Rep. 2020 Feb 19;10:2888. doi: 10.1038/s41598-020-59745-2 (PMC7031318; doi:10.1038/s41598-020-59745-2)
Supplement: Supplementary file 1 — Supplementary information [file 41598_2020_59745_MOESM1_ESM.pdf]

Supplementary Material

Title: Golden Gate assembly with a bi-directional promoter (GBid): A simple, scalable method for phage display Fab library creation

Authors: Karuppiah Chockalingam[1], Zeyu Peng[1†], Christine Vuong[2ǂ], Luc Berghman[2], Zhilei Chen[1*]

[1]Department of Microbial Pathogenesis and Immunology, Texas A&M University Health Science Center, College Station, Texas 77843, USA;

[2]Department of Poultry Science, Texas A&M University, College Station, Texas 77843, USA.

[†]Current address: Biosion, Inc., Nanjing, China 210061

[ǂ]Current address: Department of Poultry Science, University of Arkansas, Fayetteville, Arkansas 72703, USA.

Titration of phage displaying Trastuzumab Fab and V^- Fab

Phage displaying Trastuzumab Fab and V^- Fab (variable domain-lacking negative control) were produced either in the presence or absence of IPTG using the phagemids pGBid Trast Fab and pGBid, respectively, as described in the main text Methods. These phage were titered by colony enumeration following infection of SS320 *E. coli* with serially diluted phage and plating on carbenicillin LB agar as described in the main text Methods. Phage titers are reported in Table S1.

Table S1. Infectious titers of Trastuzumab Fab phage and V^- Fab phage produced in the presence or absence of IPTG.

Phage stock	# Colonies on 1e-6 plate	# Colonies on 1e-7 plate	# Colonies on 1e-8 plate	Calculated titer of stock (IU/mL)	Total virion yield
Trastuzumab Fab IPTG-	Too many	Too many	91	9.1e12	9.1e12
Trastuzumab Fab 0.05 M IPTG	52	9	N/A	5.2e10	1.0e10
Trastuzumab Fab 0.1 M IPTG	59	7	N/A	5.9e10	1.2e10
V^- Fab 0.1 M IPTG	84	7	N/A	8.4e10	1.7e10

Calculation of Phage Titer

$$\text{Phage titer} = (\#\ \text{colonies}) \times (\text{dilution factor}) \times \frac{1\ \text{mL total infected cells}}{0.1\ \text{mL plated}} \times \frac{1000\ \mu\text{L/mL}}{10\ \mu\text{L phage inoculated}}$$

Eg. If 100 colonies are counted on 1e-6 plate, then:

Phage titer = 100 x 1e6 x 10 x 100 = 1e11 infection units (IU)/mL

Evaluation of binding of Trastuzumab Fab phage produced in the presence of IPTG to HER2 ectodomain

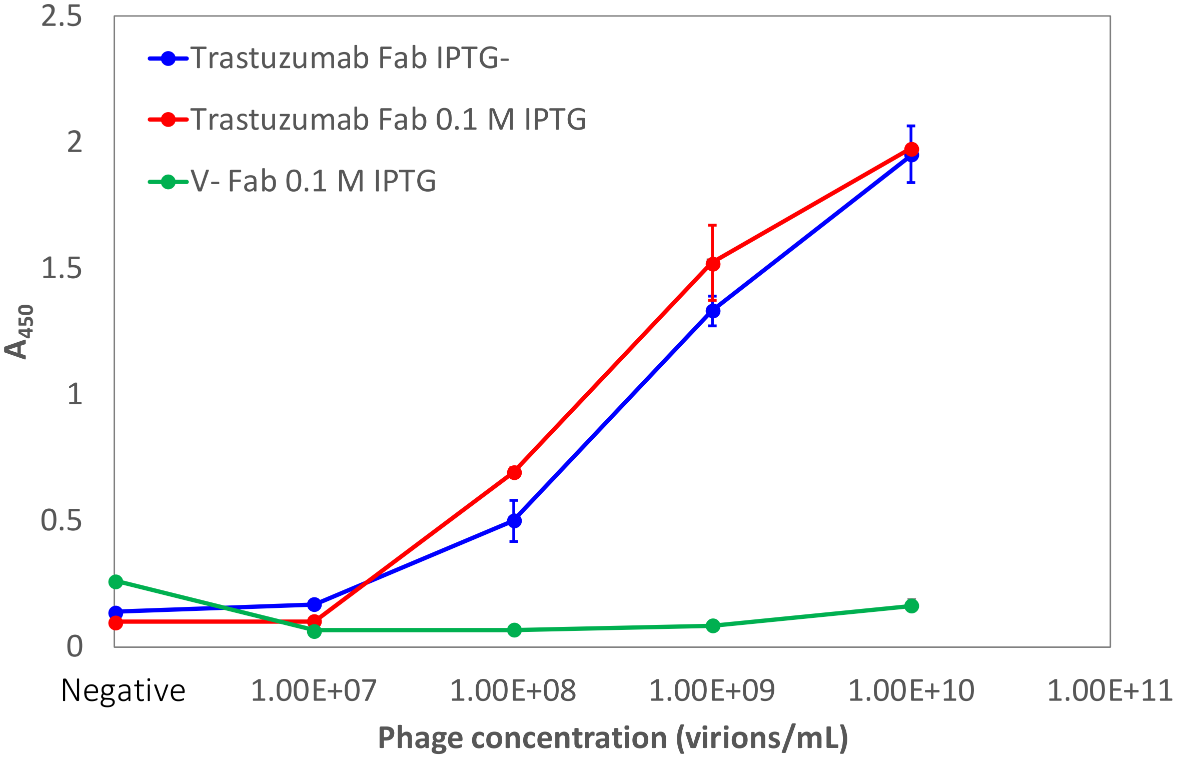

Figure S1. Induction of Trastuzumab Fab expression with 0.1 mM IPTG during M13K07 helper phage-assisted phage production using phagemid pGBid does not significantly enhance HER2 binding. Phage displaying Trastuzumab Fab or only the constant regions of the Fab (V^- Fab) produced in the presence or absence of 0.1 M IPTG were evaluated for binding to HER2 ectodomain-coated ELISA plates as described in the main text. The "Negative" condition in the X-axis refers to wells coated with DPBS instead of HER2 ectodomain and incubated with 10^{10} virions/mL. Values are the mean ± SD of duplicate measurements.

Generation of hCD20-positive HD11 cells for chicken immunization

To create the hCD20-expressing lentiviral provirus pLenti6-hCD20, the cDNA for human CD20 (MS4A1) was cloned into the *Bam*HI and *Xho*I sites of pLenti6 (ThermoFisher) downstream of the CMV promoter. Lentiviral particles pseudotyped with the envelope glycoprotein of vesicular stomatitis virus (VSV-G) were generated by co-transfection of HEK293T cells with pLenti6-hCD20, HIV gag-pol [1] and VSV-G [1]. The hCD20-VSV-G pseudoparticle-containing supernatant was collected two days post transfection, filtered through a 0.45 μm PES filter and stored at -80°C in aliquots.

Chicken macrophage-like HD11 cells (ATCC) cultured at 37°C/5% CO_2 in DMEM medium supplemented with 10% chicken serum were transduced with hCD20-VSV-G pseudoparticles. Three days post transduction, the transduced cells were selected and expanded in DMEM/10% chicken serum medium supplemented with 10 μg/mL blasticidin for 7 days and cryopreserved in liquid nitrogen. The expression of hCD20 in transduced HD11 cells was confirmed by staining these cells with mouse anti-human CD20 antibody clone 2H7 (BD Biosciences, 10 μg/mL) followed by secondary staining with 4 μg/mL DyLight 488 goat anti-mouse IgG antibody (Jackson ImmunoResearch). About 50% of the cells were found to be hCD20-positive by flow cytometric analysis.

Immunization of chickens with hCD20-displaying HD11 cells

Three layer chickens were immunized with hCD20-positive HD11 cells and one control layer chicken was immunized with naïve HD11 cells. All chickens were ~27 weeks of age at first immunization. On Day 0, each chicken received injections of 10^7 hCD20-positive HD11 or naïve HD11 cells per site at three sites – subcutaneous (SC), intramuscular (IM), and intraperitoneal (IP) – in 0.5 mL DPBS. On Day 21, each of the chickens received a booster immunization at three sites, again SC, IM and IP, this time with 10^6 hCD20-positive HD11 cells per site. Serum was collected from each animal 1 week after the booster to assess the reactivity with hCD20-positive cells (Figure S2). On Day 39, each chicken was injected intravenously with 5 x 10^6 hCD20-positive HD11 cells in DPBS. The birds were euthanized on Day 42 and the bone marrow and spleens immediately preserved in liquid nitrogen. Animal care and handling was approved by Texas A&M University Institutional Animal Care and Use Committee (permit # 2017-0353).

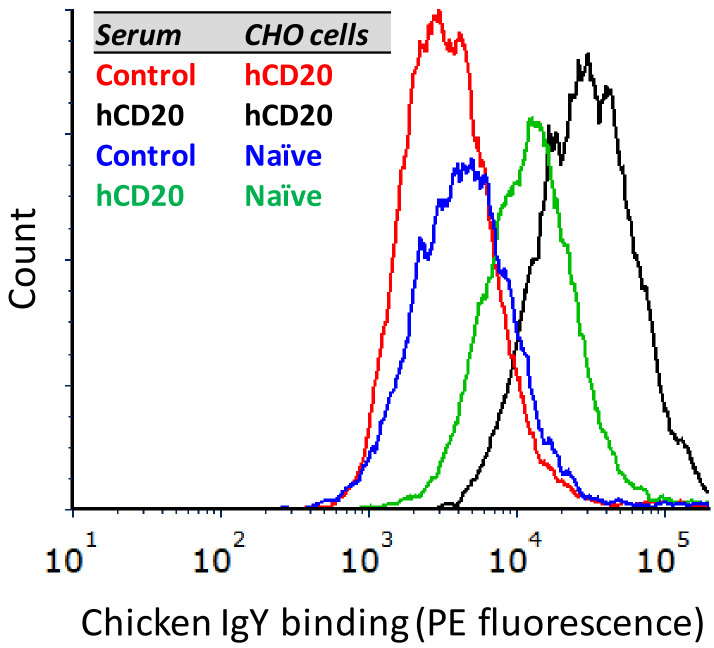

Figure S2. Reactivity of immunized chicken serum for either CHO cells stably transduced with hCD20 or naïve CHO cells as assessed by flow cytometry. Binding of chicken immunoglobulins was detected with goat anti-chicken PE secondary antibody (Millipore, AP503H) after incubation with a 1:100 dilution of chicken serum in DPBS/1% BSA. hCD20 serum: post-boost serum from chickens immunized with hCD20-positive HD11 cells; control serum: post-boost serum from chickens immunized with naive HD11 cells. While hCD20 serum is strongly reactive with the hCD20-CHO cells relative to the control serum, the same hCD20 serum is also weakly reactive with naïve CHO cells relative to the control serum. The factors causing this background reactivity are presently unclear, but could conceivably derive from an upregulation of antigenic non-hCD20 surface epitopes on the hCD20-transduced HD11 cells that are homologous to surface factors expressed on CHO cells.

Isolation of total RNA from spleen and bone marrow of immunized chickens

Spleen (~70 mg) and bone marrow (~90 mg) samples from each chicken were homogenized separately in 1 mL TRI Reagent (Sigma-Aldrich) using a Dounce tissue grinder (Wheaton). 200 μL of chloroform was mixed in with the homogenized samples followed by incubation at ambient temperature for 5 minutes. The mixtures were centrifuged at 12,000 *g* and 4°C for 15 minutes. 400 – 500 μL of the aqueous (top) phase were transferred to clean RNase-free 1.5 mL microcentrifuge tubes. An equal volume of isopropanol was added and the samples mixed and incubated at ambient temperature for 10 minutes. The precipitated nucleic acids were collected by centrifugation at 12,000 *g* and 4°C for 10 minutes. The supernatant was removed and the pellets washed with gentle vortexing using 1 mL 75% ethanol followed by centrifugation at 12,000 *g* and 4°C. The ethanol was removed and the pellets air-dried prior to resuspension in 80 μL RNase-free water. 10 μL of 10X DNase I reaction buffer and 10 μL of DNase I (Life Technologies) were added to each sample followed by incubation at ambient temperature for 15 minutes. 400 μL RNase-free water and 500 μL acid-phenol/chloroform were added followed by mixing by inversion and centrifugation at 10,000 *g* and 4°C for 5 minutes. 400 μL of the aqueous phase was transferred to a new tube and the nucleic acids precipitated by addition of 40 μL 3 M sodium acetate, pH 5.5 and 1 mL 100% ethanol. The samples were mixed by gentle inversion and incubated at -20°C for 30 minutes. The RNA was pelleted by centrifugation at 20,000 *g* and 4°C for 15 minutes. The supernatant was removed and the RNA pellets washed twice with 1 mL 75% ethanol with centrifugation at 12,000 *g* and 4°C for each wash step. The ethanol was removed and the pellets air-dried. The RNA pellets were solubilized in 30 μL RNase-free water with brief incubation at 55°C. RNA concentrations were quantified using a Nanodrop 1000 spectrophotometer (Thermo Scientific) and the samples stored at -80°C.

Synthesis of first-strand cDNA

The SuperScript First-Strand Synthesis System for RT-PCR (Life Technologies) was used according to the manufacturer's recommendations. Briefly, for each spleen or bone marrow total RNA sample, 5 μg RNA was incubated with 1 mM dNTP mix, 50 ng/μL oligo(dT)$_{12-18}$ primer and DEPC-treated water in a 10-μL final volume at 65°C for 5 minutes followed by incubation on ice for at least 1 minute. A 2X reaction mixture containing 2X RT buffer, 10 mM $MgCl_2$, 20 mM DTT, and 4 U/μL RNaseOUT was separately prepared and 9 μL of the 2X reaction mixture added to the RNA/primer mixture followed by mixing and incubation at 42°C for 1 minute. 1 μL SuperScript II Reverse Transcriptase was added to each tube and the reactions were incubated at 42°C for 50 minutes. The reactions were terminated at 70°C for 15 minutes followed by addition of RNase H and incubation at 37°C for 20 minutes. The completed reactions were stored at -20°C.

Amplification of chVL and chVH library fragments

The chVL and chVH chains were separately amplified using either spleen- or bone marrow-derived first-strand DNA pooled from three immunized chickens as a template using the primer pairs chVL-F/chVL-R and chVH-F/chVH-R (Table 1, main text). Eight 50-μL high-fidelity PCR amplifications were performed for each template and primer-pair combination (32 reaction total). Each reaction contained 1 μL first-strand cDNA template, 0.2 mM dNTPs, 1X Phusion HF buffer, 0.5 μM each primer, and 1 unit Phusion DNA Polymerase. Amplifications were performed in a Bio-Rad C1000 Touch Thermal Cycler using the following program for spleen first-strand cDNA-templated reactions: 98°C 30 s, followed by 35 cycles of 98°C 10 s, 60.5°C 15 s, 72°C 15 s, followed by 72°C 5 mins. Bone marrow first-strand cDNA amplifications were performed the same way except an annealing temperature of 63.8°C was used instead of 60.5°C. The amplified VL and VH chains were agarose gel-purified and the purified fragments from the different sources (spleen and bone marrow) pooled.

Generation and cloning of a Fab library insert, chVL-BidP-chVH, derived from hCD20-immunized chickens

Overlap extension PCR of the chVL, BidP, and chVH fragments was performed as described in the main text, yielding a dominant chVL-BidP-chVH fragment of the correct size (1070 bp) accompanied by additional non-specific products that manifested as a smear in agarose gel electrophoresis (Figure S3A). The chVL-BidP-chVH amplification reaction containing the non-specific fragments was cleaned up using the QIAquick PCR Purification kit (Qiagen) and used in a Golden Gate reaction with the pGBid destination vector as described in the main text. The ~1 kb fragment of interest was quantified by comparison with a DNA marker in an agarose gel analysis. Colony PCR [2] was carried out on randomly selected transformants using the flanking primers chVL-R and chVH-R to gauge the proportion of clones carrying the full-length chVL-BidP-chVH insert, indicating 15 out of 19 clones (78.9%) with the correct 1070 bp insert (Figure S3B).

A

B

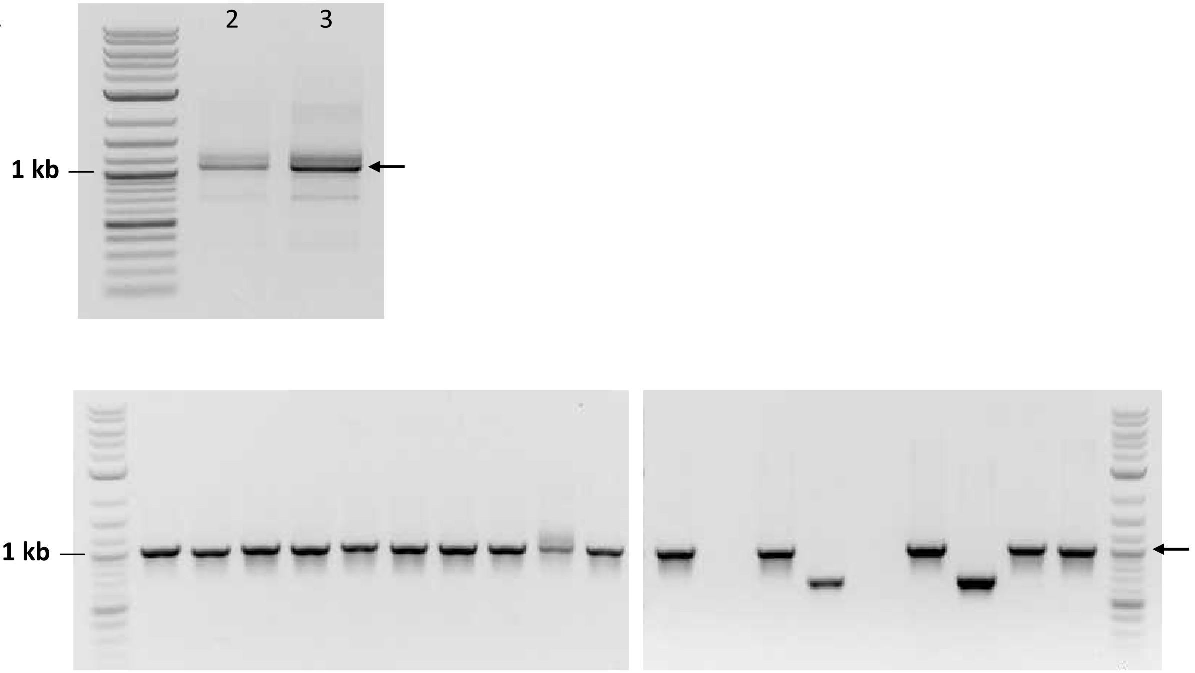

Figure S3. Generation and cloning of a Fab library insert, chVL-BidP-chVH, derived from hCD20-immunized chickens. (A) Agarose gel electrophoresis of the crude chVL-BidP-chVH product created by overlap extension (OE) PCR. Lane 2: 0.5 μL load; lane 3: 1.0 μL load. (B) Colony PCR analysis of 19 randomly selected library transformants after Golden Gate incorporation of the non-gel-purified chVL-BidP-chVH product into pGBid. The arrows indicate the expected size of the full-length chVL-BidP-chVH insert.

Colony PCR of phagemid clones within Round 2 output

To evaluate the proportion of phagemid-containing clones in Round 2 of anti-hCD20 Fab whole-cell panning that contain full-length chicken-human Fab inserts, individual phagemid-containing colonies from selection Round 2 were subjected to colony PCR [2] using the primers chVL-R and chVH-R (Table 1, main text). Specifically, *E. coli* cultures containing amplified phage recovered from selection Round 2 were plated on LB agar plates containing 50 μg/mL carbenicillin and 20 well-isolated colonies were selected for growth in 50 μg/mL carbenicillin-supplemented LB medium overnight. 2 μL of the overnight cultures were used as a template for 20-μL PCR reactions containing 1X Standard Taq Buffer, 0.2 mM dNTP mix, 0.5 μM primers chVL-R and chVH-R, and 0.5 units Taq DNA Polymerase (NEB). The following thermal cycling program was used: 96°C 45 s, followed by 35 cycles of 95°C 15 s, 58°C 15 s, 68°C 70 s, and a final extension at 68°C for 5 minutes. 5 μL of the completed reactions were electrophoresed on an agarose gel at 120 V for 23 minutes and the gel imaged, indicating a predominance of incorrect inserts or no insert at all (Figure S4). The tendency for insert-free phagemid to be selected during whole-cell phage panning, presumably due to the reduced metabolic burden imposed by the insert-free phagemid on the host *E. coli* cells, has been observed previously [3, 4]. An approach to minimize this undesirable phenomenon has been to apply stringent washing during panning using low-pH (pH 5.0) buffer. It is noted that each round of phage panning in this study included three washes with pH 5.0 buffer.

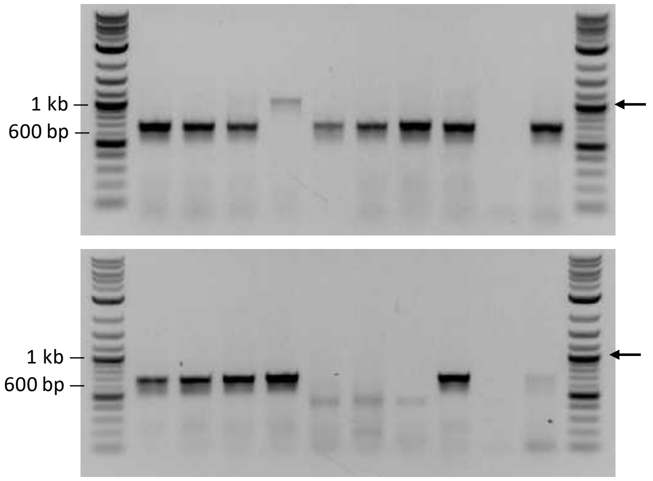

Figure S4. Colony PCR of chVL-BidP-chVH inserts within monoclonal phage enriched in Round 2 of whole-cell panning for hCD20 binders, indicating a predominance (19/20 or 95%) of clones lacking the full-length insert. The expected size of the full-length chVL-BidP-chVH insert is approximately 1 kb (indicated by arrows).

Re-cloning of full-length chVL-BidP-chVH inserts from Round 2 using GBid

Agarose gel purification was used to recover and enrich the small fraction of full-length chVL-BidP-chVH inserts present in the Round 2 phagemid pool. Briefly, the cells from *E. coli* cultures containing amplified phage recovered from selection Round 2 were pelleted by centrifugation and the phagemid extracted using the EZNA Plasmid Mini Kit I (Omega Bio-tek). 14 μg of the purified phagemid pool was used in a restriction digestion reaction containing 140 units of each of the enzymes *Sna*BI and *Sac*I-HF, carried out at 37°C for 3 h according to the recommendations of the manufacturer NEB. The entire reaction was electrophoresed on a 1% agarose gel at 100 V for 30 minutes and a 1.2 kb fragment containing the full-length chVL-BidP-chVH insert pool was excised and extracted using the Zymoclean Gel DNA Recovery Kit (Zymo Research). 10 ng of this purified full-length Round 2-enriched chVL-BidP-chVH insert pool was used as a template in each of 12 50-μL high-fidelity PCR amplifications containing 1X Phusion HF Buffer, 0.2 mM dNTP mix, 0.5 μM primers chVL-R and chVH-R, and 1 unit Phusion DNA Polymerase (NEB). Twenty cycles of amplification were performed according the thermal cycling scheme described for GBid in the main text. The PCR product was cleaned up and concentrated to a final volume of 100 μL using the QIAquick PCR Purification Kit (Qiagen). The concentrated product was electrophoresed through a 1% agarose gel at 100 V for 30 minutes and the ~1.1 kb chVL-BidP-chVH insert excised and extracted as described above, yielding ~60 μL of purified product at ~100 ng/μL.

2.44 μg of the purified enriched Round 2 full-length insert pool was used as an insert in a 3-mL Golden Gate assembly set up according to the optimized conditions described in the main text. The completed Golden Gate reaction was concentrated using a centrifugal filter and ethanol precipitation as described in the main text and the DNA pellet solubilized in 50 μL high-purity water. 20 μL of this full-length-insert-enriched Round 2 phagemid pool was used in a single electroporation in a chilled 2-mm gap cuvette at 2500 V containing 380 μL electrocompetent SS320 *E. coli* followed by recovery in 20 mL prewarmed SOC medium. Phage was generated as described for the initial library phage pool in the

main text with volumes linearly scaled down as appropriate for the single electroporation. A small amount of the electroporated cells was plated on carbenicillin-containing LB agar plates for enumeration of transformants, indicating 2.3 x 10^9 independent transformants in the re-cloned Round 2 library. Colony PCR was carried out as described above on 20 randomly selected phagemid-containing colonies, indicating that 100% of the re-cloned Round 2 phagemid pool contain full-length chVL-BidP-chVH inserts (Figure S5). The re-cloned phage pool was used for the 3rd round of whole-cell phage panning.

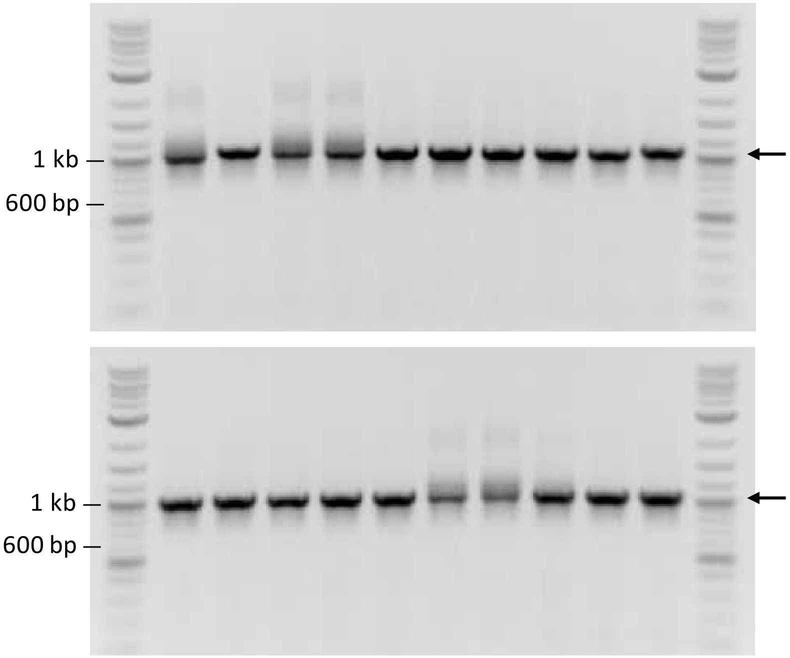

Figure S5. Colony PCR analysis of chVL-BidP-chVH inserts within the Round 2 phagemid pool constructed by re-cloning only full-length inserts from the crude whole-cell panning Round 2 phage output. Colony PCR was performed on the re-cloned 2nd-round phagemid pool as described above under the subheading "Colony PCR of phagemid clones within Round 2 output”. In contrast to Figure S4 which indicates only 5% (1/20) representation of the correct ~1 kb full-length chVL-BidP-chVH insert in the crude Round 2 phage output, the re-cloned phagemid pool exhibits 100% (20/20) full-length insert representation. The arrows indicate the expected size of the full-length chVL-BidP-chVH insert.

Isolation of rare non-Fab AC1 hCD20 binders

Colony PCR and sequencing of the VH chain of 20 randomly selected phagemid clones from the 4th-round selected phage pool revealed a single full-length unique sequence. Two VL chains were sequenced from among these 20 clones and both light chains were also identical, suggesting that the 4th-round phage pool was dominated by a single clone, named Fab AC1. In an effort to identify hCD20-specific non-Fab AC1 clones, 46 randomly selected monoclonal phagemids from the 3rd-round selection pool were subjected to sequencing and flow cytometry. Surprisingly, 28 (~60%) of these 3rd-round clones did not express full-length Fabs due to the presence of nucleotide insertions/deletions that introduce frameshifts in the open reading frame of the coding sequence and/or premature stop codons; of the 18 remaining fully expressed Fabs, five specifically bound hCD20-transfected cells and all five had the same or a similar VH chain to Fab AC1. Thirteen unique full-length Fabs with no sequence resemblance to Fab AC1 were identified amongst the 46 selected 3rd-round clones, but none exhibited specific binding to hCD20-transfected CHO cells.

While non-Fab AC1 Fabs could not be identified from sequencing 20 randomly selected 4th-round clones, we hypothesized that rare non-Fab AC1 hCD20-specific clones might still exist within the heavily hCD20-selected 4th-round phagemid pool and therefore sought to selectively deplete the 4th-round phagemid pool of Fab AC1. Further scrutiny of the VH sequence of Fab AC1 revealed the presence of two restriction enzyme recognition sites – *Nco*I and *Ppu*MI – within the hypervariable CDR1 and CDR3 regions of the VH chain, respectively, that are not present anywhere else within the Fab insert. In order to deplete the 4th-round phagemid pool of the dominant clone and thus potentially reveal non-Fab AC1 hCD20-binders, the pool was digested separately with *Nco*I and *Ppu*MI and the digested mix was either used to directly transform SS320 *E. coli* (*Nco*I-treated pool) or used as a template to PCR-amplify any remaining undigested Fabs (*Ppu*MI-treated pool). The PCR product enriched for Fabs without the *Ppu*MI site was re-cloned into pGBid via Golden Gate assembly and this reaction was used to transform SS320

E. coli cells as described in the main text. 36 monoclonal 4th-round phagemids selected from the *Nco*I- and *Ppu*MI-treated pools depleted of clone Fab AC1 were digested again with either *Nco*I or *Ppu*MI and analyzed via agarose gel electrophoresis to confirm removal of the sites. Clones showing removal of the Fab AC1-contained *Nco*I/*Ppu*MI site (approximately half of those selected for analysis) were sequenced, confirming that none were the dominant 4th-round clone Fab AC1. Phage were produced from these clones and the ability of the monoclonal phage to bind EGFP-hCD20-transfected CHO cells was evaluated by flow cytometry as indicated in the main text. One completely unique clone having a distinct VH and VL to Fab AC1 was represented several times within the restriction enzyme-treated pools and showed specific binding to EGFP-hCD20-transfected CHO cells, Fab AC11. Many non-Fab AC1 clones within the Fab AC1-depleted pool that exhibited binding to EGFP-hCD20-CHO cells shared the same or a similar VH chain to Fab AC11 but had a different VL chain.

Colony PCR analysis of 3rd-round and 4th-round phagemid outputs

To gauge the percentage of phagemids containing full-length chVL-BidP-chVH inserts in the 3rd-round and 4th-round selection outputs, colony PCR was carried out as described above under the subheading "Colony PCR of phagemid clones within Round 2 output" (Figure S6).

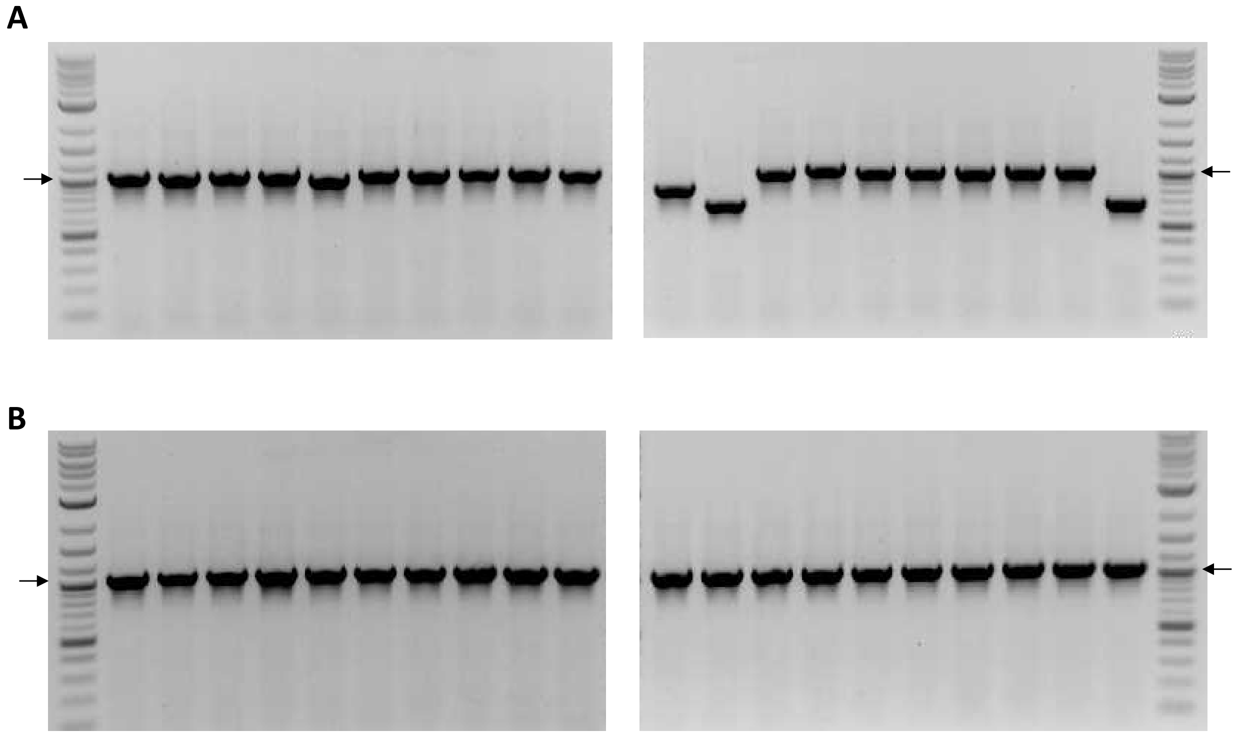

Figure S6. Colony PCR of chVL-BidP-chVH inserts in 20 randomly selected phagemid clones from the selection outputs of panning Round 3 (A) and Round 4 (B), indicating 17/20 (85%) and 20/20 (100%) full-length Fab inserts, respectively. The arrows indicate the expected size of the full-length chVL-BidP-chVH insert.

Supplementary References

1. Evans, M.J., et al., *Claudin-1 is a hepatitis C virus co-receptor required for a late step in entry.* Nature, 2007. **446**(7137): p. 801-5.
2. Woodman, M.E., *Direct PCR of intact bacteria (colony PCR).* Curr Protoc Microbiol, 2008. **Appendix 3**: Appendix 3D.
3. Jones, M.L., et al., *Targeting membrane proteins for antibody discovery using phage display.* Sci Rep, 2016. **6**: p. 26240.
4. Tur, M.K., et al., *Selection of scFv phages on intact cells under low pH conditions leads to a significant loss of insert-free phages.* Biotechniques, 2001. **30**(2): p. 404-8, 410, 412-3.
